# Prognosis and outcome of intensive care for very elderly patients

**DOI:** 10.1186/2197-425X-3-S1-A367

**Published:** 2015-10-01

**Authors:** S Becker, J Müller, G de Heer, V Fuhrmann, S Kluge

**Affiliations:** Intensivmedizin, Universitätsklinikum Hamburg Eppendorf, Hamburg, Germany

## Introduction

The proportion of elderly and very elderly patients is rising in industrial countries. Elderly patients are expected to outnumber younger age groups in the use of health services in the future. Since the prognosis of elderly patients is generally considered to be poor, the indication for ICU admission is kept very strict.

## Objectives

The aim of this study was to analyze the characteristics and outcome of intensive care treatment of very elderly patients (≥90 years), as this age group is growing rapidly and is becoming of increasing importance in intensive care medicine.

## Methods

Monocentric, retrospective evaluation of patients aged ≥ 90 years admitted to the intensive care units at the Hamburg University-Hospital between 01.01.2008 and 30.6.2013.

## Results

A total of 372 patients ≥ 90 years were admitted to the intensive care units (ICUs). The majority (66.7%) of patients were admitted because of an emergency, and of which, half underwent unscheduled surgery. 39.8% needed support by mechanical ventilation and vasoactive drugs, 1,9% received renal replacement.

ICU and hospital mortality rates were 18.3% and 30.9%, respectively. Mortality at one year after hospital discharge was 46.3%.

Binary regression analysis revealed high SAPS II scores on admission and necessity of mechanical ventilation as independent risk factors for ICU-mortality.

17.5% of patients had an advance directive.

## Conclusions

Nearly 70% of patients aged ≥ 90 years were discharged alive from hospital following treatment at the ICU, more than half were still alive one year after discharge. Nowadays, age no longer determinates admission to intensive care.

